# Interventions to improve physical activity among socioeconomically disadvantaged groups: an umbrella review

**DOI:** 10.1186/s12966-018-0676-2

**Published:** 2018-05-15

**Authors:** Melinda Craike, Glen Wiesner, Toni A. Hilland, Enrique Garcia Bengoechea

**Affiliations:** 1 Institute for Health and Sport, Victoria University, PO Box 14428, Melbourne, VIC 8001 Australia; 2 School of Education, College of Design and Social Context, RMIT, PO Box 71, Bundoora, VIC 3083 Australia; 3 Department of Physical Education and Sport Sciences, Faculty of Education and Health Sciences, University of Limerick, Limerick, Ireland

**Keywords:** Physical activity, Intervention, Socioeconomic disadvantage, Adults, Children, Adolescents, Effectiveness, Underserved, Impoverished

## Abstract

**Background:**

People from socioeconomically disadvantaged population groups are less likely to be physically active and more likely to experience adverse health outcomes than those who are less disadvantaged. In this umbrella review we examined across all age groups, (1) the effectiveness of interventions to improve physical activity among socioeconomically disadvantaged groups, (2) the characteristics of effective interventions, and (3) directions for future research.

**Methods:**

PubMed/MEDLINE and Scopus were searched up to May 2017 to identify systematic reviews reporting physical activity interventions in socioeconomically disadvantaged populations or sub-groups. Two authors independently conducted study screening and selection, data extraction (one author, with data checked by two others) and assessment of methodological quality using the ‘Assessment of Multiple Systematic Reviews’ scale. Results were synthesized narratively.

**Results:**

Seventeen reviews met our inclusion criteria, with only 5 (30%) reviews being assessed as high quality. Seven (41%) reviews focused on obesity prevention and an additional four focused on multiple behavioural outcomes. For pre school children, parent-focused, group-based interventions were effective in improving physical activity. For children, school-based interventions and policies were effective; few studies focused on adolescents and those that did were generally not effective; for adults, there was mixed evidence of effectiveness but characteristics such as group-based interventions and those that focused on physical activity only were associated with effectiveness. Few studies focused on older adults. Across all ages, interventions that were more intensive tended to be more effective. Most studies reported short-term, rather than longer-term, outcomes and common methodological limitations included high probability of selection bias, low response rates, and high attrition.

**Conclusions:**

Interventions can be successful at improving physical activity among children from socioeconomically disadvantaged groups, with evidence for other age groups weak or inconclusive. More high-quality studies in this population group are needed, which adopt strategies to increase recruitment rates and reduce attrition, report longer term outcomes, and provide adequate intervention details, to allow determination of the characteristics of effective interventions. We recommend that the benefits of physical activity be recognised more broadly than obesity prevention in future studies, as this may have implications for the design and appeal of interventions.

**Electronic supplementary material:**

The online version of this article (10.1186/s12966-018-0676-2) contains supplementary material, which is available to authorized users.

## Background

In developed nations, physical activity and associated health outcomes are socially distributed and vary by socioeconomic position [1]. People who are socioeconomically advantaged are more likely to meet recommended levels of participation in physical activity and less likely to experience adverse health outcomes associated with inactive lifestyles, than their less advantaged peers [2, 3]. The substantial socioeconomic gradient in participation in physical activity has been observed across all age groups, starting in early childhood [4–6]. Despite recommendations for action on the social determinants of health dating back to the 1980s, inequalities in many countries continue to grow and are now a widely recognised problem that requires immediate and significant action [7].

Improving participation in physical activity in socioeconomically disadvantaged population groups is a public health challenge. Most interventions aimed at improving physical activity have been developed and evaluated in the general population with little regard for their impact across social strata [8, 9]. However, interventions which do not consider the special needs and barriers of socioeconomically disadvantaged groups may be less effective [10, 11]. It has been shown, for example, that population-wide public health strategies can be differentially effective between socioeconomic groups and even increase inequalities [12]. Hence, it is important that physical activity interventions target socioeconomically disadvantaged groups or examine the effectiveness of interventions across social strata.

Several systematic reviews have examined the effectiveness of interventions to improve physical activity among socioeconomically disadvantaged groups (e.g. [13, 14]). However, to date, there has been no synthesis of the findings of these reviews. We conducted an umbrella review to integrate the findings of systematic reviews across all age groups. Specifically, the aims of this umbrella review were to: (1) examine the effectiveness of interventions to improve participation in physical activity among socioeconomically disadvantaged groups, (2) examine the characteristics of effective interventions, and (3) provide recommendations for future research.

## Methods

### Main search strategy

PubMed/MEDLINE and Scopus were searched up to May 2017 with no date limitation to identify systematic reviews and meta-analyses. Groups of thesaurus terms and free terms for ‘physical activity’ (e.g. sport, walking, exercise), ‘interventions’ (e.g. trial, program, implementation), ‘social disadvantage’ (e.g. low socio-economic status, low income, underserved) and publication type (e.g. meta-analysis, review) were used. This resulted in the following example search: title-abs-key(“physical* activ*” OR sport* OR walking OR exercise OR lifestyle OR “life style” OR “physical fitness” OR “motor activi*”) AND title-abs-key(“low SES” OR “low* socio*” OR “low* income” OR disadvantaged OR inequal* OR disparity OR deprived OR underserved OR “low* educat*” OR poverty OR “social class” OR equity) AND title-abs-key(RCT OR intervention OR program* OR implementation OR evaluation OR trial) AND title-abs-key (review OR meta-analysis OR synthesis). Reference lists of all included papers were manually checked to identify additional relevant articles.

To be included in our umbrella review, articles had to be written in English and evaluate physical activity promotion interventions that were either, a) targeted at disadvantaged populations or, b) universal interventions that included a sub-group analysis with a socioeconomically disadvantaged population. In addition, reviews had to evaluate at least three primary studies fulfilling the above criteria. We included systematic reviews and comprehensive reviews with a systematic search strategy. We took a broad definition of ‘intervention’ and allowed any intervention (including policies) where physical activity behaviour change was a primary or secondary objective. We also included any study design to ensure a wide variety of interventions strategies were captured.

We excluded populations characterized by chronic disease, pregnant women, or other special conditions. Hence, obesity and cardiovascular prevention interventions were considered, but not treatment interventions.

Socio economically disadvantaged population groups are generally defined as those described as low socioeconomic status, low income, low education, or from areas defined as socio economically disadvantaged (often characterized by low income levels) [15, 16]. However, there is no universally accepted definition of ‘socio economic disadvantage’ and the cut points that define socio economic disadvantage differ between studies [17]. Given these disparities, we accepted the review’s definition of socio economic disadvantage. That is, if a review described a population group as socioeconomically disadvantaged, we included it.

Titles and abstracts of the identified articles were reviewed by two authors (GW and TAH or EGB) to exclude articles out of scope. Subsequently, two authors (GW and TAH or EGB) independently reviewed the full text of all potentially relevant articles for eligibility. Disagreements between reviewers were resolved by consensus approach with a third reviewer (MC).

### Data extraction

Data extraction was conducted by one researcher (GW), with all data checked by two other researchers (TAH and EBG). Where reviews covered multiple populations, intervention foci and behavioural outcomes, the extracted data were based on and limited to the key inclusion criteria stated above. Where possible, data were extracted based on age groupings and we also attempted to extract data on the characteristics of interventions that were related to effectiveness. Thus, we not only looked at the effectiveness of community-based interventions per se, but we also attempted to identify the types and components of more effective interventions. Detailed results are included in Additional file 1. In summarising the evidence, we placed more weight on outcomes of reviews that (1) included a greater number of primary studies; and (2) were of higher quality [18].

### Quality assessment

The methodological quality of each systematic review was assessed using the ‘Assessment of Multiple Systematic Reviews’ (AMSTAR) rating scale [19]. The final item was modified to assess only the review itself given that PRISMA does not require a conflict of interest assessment for each primary study. The included systematic reviews were assessed by one researcher (GW) with all ratings checked by another researcher (EBG); disagreements were resolved by a consensus approach.

## Results

### Characteristics of included reviews

**Fig. 1:**
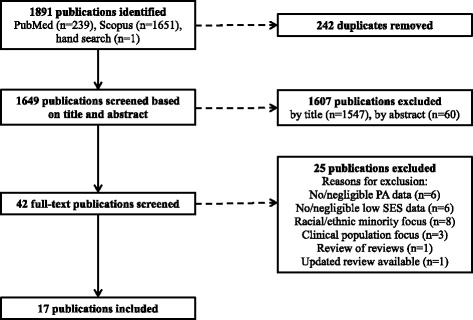
PRISMA flowchart

Forty-two full papers were assessed for eligibility, and 17 reviews were selected for synthesis (see Fig. 1 PRISMA flowchart). The main reasons for reviews being ineligible were having a broad focus on racial or ethnic minorities rather than socio economic disadvantage per se (*n* = 8) and including no or negligible number of original studies (i.e. < 3) reporting on physical activity outcomes (*n* = 6) or socio-economic disadvantage (*n* = 6).

Many reviews provided vague descriptions of the included studies, making it difficult to determine precisely which type of study designs were included. Eleven of the included systematic reviews included data from primary studies using any type of study design, often described as ‘any intervention’ [9, 14, 15, 20–27]; five were limited to controlled trials [17, 28–31] and one included only randomized controlled trials [13]. Studies that met our criteria in each review ranged from 3 to 27.

Table 1 summarises the AMSTAR review quality ratings. From a total possible score of 11, 5 (30%) of the reviews were rated 8 or above and could be considered high quality; 9 (53%) were rated 4 to 7 and could be considered medium quality; and 3 (18%) were rated as less than 4 and could be considered low quality. The ratings were partly a function of date of publication - reviews published prior to 2014 had a mean score of 4.4 (*n* = 7) and reviews published from 2014 and onwards had a mean score of 6.3 (*n* = 10). This is likely due to the more recent development and adoption of review guidelines (e.g. PRISMA and AMSTAR itself).

**Table 1:** Methodological quality assessment of included systematic reviews using AMSTAR

Author (Year)	1	2	3	4	5	6	7	8	9	10	11^a^	Rating
Bock et al. 2014 [31]	0	0	1	0	0	1	0	NA	1	0	1	4
Bull et al. 2014 [13]	0	1	1	0	0	1	1	1	1	1	1	8
Chaudhary & Kreiger 2007 [22]	0	0	0	0	0	1	NA	NA	NA	0	0	1
Cleland et al. 2012 [14]	0	0	1	0	0	1	1	1	0	0	1	5
Cleland et al. 2013 [28]	0	1	1	0	0	1	1	1	1	1	1	8
Everson-Hock et al. 2013 [23]	0	0	1	1	0	1	1	0	0	0	1	5
Kader et al. 2015 [30]	0	0	1	0	0	1	1	1	0	0	1	5
Kornet-van der Aa et al. 2017 [20]	1	1	1	0	0	1	1	1	1	0	1	8
Laws et al. 2014 [15]	1	0	1	0	0	1	1	1	1	0	1	7
Lehne & Bolte 2017 [9]	1	1	1	0	0	1	1	0	1	0	1	7
Magnee et al. 2013 [24]	0	0	0	0	0	1	1	0	1	0	1	4
Olstad et al. 2016 [25]	1	1	1	0	0	1	1	1	1	0	1	8
Olstad et al. 2017 [17]	1	1	1	0	0	1	1	1	1	0	1	8
Taylor et al. 1998 [21]	0	0	1	0	0	1	0	0	0	0	0	2
van Sluijs et al. 2007 [26]	0	0	1	0	0	1	1	1	1	0	1	6
Walton-Moss et al. 2014 [27]	0	0	0	0	0	1	0	0	1	0	1	3
Wijtzes et al. 2017 [29]	0	0	1	0	0	1	1	0	1	0	1	5

AMSTAR items were scored as “Yes” (1), “No” (0), “Can’t Answer” or “Not Applicable” (NA). AMSTAR comprises the following items:

1. ‘a priori’ design provided;

2. duplicate study selection/data extraction;

3. comprehensive literature search;

4. status of publication as inclusion criteria (i.e., grey or unpublished literature);

5. list of studies included/excluded provided;

6. characteristics of included studies documented;

7. scientific quality assessed and documented;

8. appropriate formulation of conclusions (based on methodological rigor and scientific quality of the studies);

9. appropriate methods of combining studies (homogeneity test, effect model used and sensitivity analysis);

10. assessment of publication bias (graphic and/or statistical test); and

11. conflict of interest statement*’

^a^Criterion modified to solely asses conflict of interest/source of funding statement of the review

### Evidence relating to interventions to improve physical activity by age group

#### Children and adolescents

Eight reviews included separate data on children and adolescents (aged under 18 years) with several focusing on specific age groups. Two recent and medium quality reviews focussing on pre-school children suggest that interventions targeting parents are likely to be effective in improving pre-school children’s physical activity [15, 30]. One of these focused specifically on pre-schoolers aged less than 5 years and examined interventions in the home setting, primary health care setting, preschool setting, and community setting. Of the 11 included studies that examined physical activity outcomes, six showed a significant effect and five showed no effects; notably all three community-based interventions were effective [15]. The other review of parental focused interventions found that two out of three studies (two community, one pre-school based) with parents of pre-school children from low socio-economic groups were effective in improving physical activity, with the remaining study showing no effect and a high dropout rate [30]. These reviews reported that most of the included studies were of low or moderate quality, and thus more high-quality studies in this age group are needed. The reviews noted that methodological limitations of the studies included physical activity measures which had not been validated, a high probability of selection bias due to the methods of recruitment, a low proportion of eligible participants who agreed to participate, and high attrition [15, 30]. One of the reviews also noted that few studies reported follow-up measures of more than 6 months, and thus the longer- term impact of interventions could not be ascertained [30].

Three reviews that included data on children from a broad age range (generally less than 12 years) found that physical activity interventions, particularly those that were school-based and multicomponent were likely to be effective [17, 25, 26]. Two of these reviews were recent and high-quality [17, 25] and examined the impact of policies on obesity-related behaviours. One of these reviews [17] found that two targeted policies (out of six) were effective in improving physical activity among children, one was organisational and one governmental. Additionally, one organisational policy aimed at school children had mixed results, indicating there was no effect for accelerometry outcomes but positive outcomes for sports participation. The review noted that most studies were of strong or moderate quality and were conducted over the longer- term. The other review, which examined the impact of universal policies (i.e. those targeting the entire population) on obesity-related behaviours in disadvantaged populations [25], found that two policies (out of 4) showed a positive impact on children’s physical activity levels. One of these policies was a provincial school physical education policy requiring students to take physical education to graduate from secondary school and the other was a children’s fitness tax credit. Overall, based on these reviews, there was some evidence for the effectiveness of comprehensive interventions that included school policies, and for government policies targeting children in school settings. Common elements of successful policy-focused interventions included enhancements to physical education, additional physical activity opportunities, school self-assessments, and education about physical activity. The third review was over 10 years old and of medium quality and found that all three school-based interventions, mostly of high quality, reported significant positive effects [26]. Each of the three reviews noted that it was challenging to determine the characteristics of effective interventions due to the multi-component nature of the interventions and inadequate descriptions of the interventions.

Six of the included reviews of children focused on obesity prevention or included behaviours aimed at preventing overweight or obesity [15, 17, 20, 25, 29, 30]. These reviews included outcomes such as body mass index (BMI) along with physical activity and dietary behaviours. Notably, but perhaps not surprisingly, obesity prevention interventions where increasing physical activity was the primary behavioural target tended to report greater improvements in participation in physical activity compared to interventions where physical activity was a secondary target [15, 29]. For example, a recent, medium quality, review included five relevant studies [29] and examined the effectiveness of interventions to improve lifestyle behaviours and/or prevent overweight among socioeconomically disadvantaged primary school aged children in Europe. This review found that all three studies that included physical activity as the primary outcome were effective. By contrast, neither of the two weight prevention-focused interventions were effective for physical activity change, which was assessed as a secondary outcome. The three effective physical activity focused interventions used the school curriculum as a delivery channel, complemented in some cases with extracurricular activities which included encouragement of physical activity outside of school hours, attendance at local sports clubs; accessible school sports activities offered on a daily basis during out-of-school hours, recurrent breaks for physical activity, relaxation exercises, and posture exercise during regular lessons [29].

Three reviews included results specifically relating to adolescents [14, 20, 26]. These reviews found that interventions were not effective in improving physical activity among this age group. A recent, high quality review which included five relevant studies [20] found that none of the interventions, nearly all of which were multi-component, combining behavioural, educational and/or environmental components, reported significant improvements in engagement in physical activity among socio economically disadvantage adolescents The authors noted that all five studies were moderate-high quality [20]. Another high-quality review showed that only two of six group-based interventions that targeted children and adolescents improved physical activity and thus concluded that group-based interventions were unlikely to improve physical activity in this age group [14]. An older, medium quality reported that only one of two intervention studies aimed at low socioeconomic adolescents reported a significant intervention effect. Consequently, evidence of an effect was deemed inconclusive [26].

#### Adults

Data on adults (18 years and over) could be extracted from nine reviews [13, 14, 17, 21, 23, 25, 27, 28, 31].

Findings from high quality reviews showed that evidence of the effectiveness of interventions was mixed and therefore inconclusive (e.g., [13, 17, 25, 31]). Furthermore, several reviews stated that the characteristics of effective interventions could not be identified due to insufficient descriptions of the intervention components, few studies meeting eligibility criteria and the multi-component nature of the included interventions [13, 14, 17, 25, 27, 31].

A high-quality review [31] that included only controlled studies found that four out of the eight studies that focused on disadvantaged groups reported improvements in physical activity, although the mean percentage change was low and insignificant (net percent change 7.7% 95% CI − 6.7% to 22.0%; *p* = 0.248). A high-quality review of behavioural interventions that included only RCTs [13], which included 12 relevant studies, found that post-intervention effects were positive but small for physical activity (standardized mean difference [SMD] 0.21, 95% CI 0.06 to 0.36). Longer- term data were available for three studies, which showed that intervention effects were not maintained at follow-up, which ranged from 6 to 8 months post baseline (SMD 0.17, 95% CI − 0.02 to 0.37). A lower quality and older review [21] found that out of eight studies with physical activity data, only two interventions reported consistent and positive physical activity changes; two showed mixed results and two were positive for specific sub-groups only (e.g. community coalitions or organised communities). A further two reviews focusing on the effectiveness of policies in improving physical activity and reducing inequality suggested that gaps in knowledge remain surrounding effective physical activity policies in adults [17, 25]. A recent, high-quality review of the impact of targeted policies on obesity-related behaviors found only one policy related to adults, a government policy implemented in community settings, which did not have an impact on physical activity behavior. The review concluded that there was a general absence of high-quality evidence pertaining to the impact of targeted policies outside of school environments [17]. A second recent, high-quality review which included three studies of adults noted that for all of the obesity-related behaviours examined, including physical activity, no clear patterns of policies that positively or negatively impacted inequities could be discerned, with most universal polices having a neutral impact. Notably, no policies negatively impacted inequities [25].

Two reviews found that interventions solely targeting physical activity were more effective than interventions that targeted multiple behaviours, such as diet plus physical activity [13, 23]. For example, Bull et al. [13] found that effects were larger (*p* < 0.001) in the seven interventions targeting physical activity only (SMD 0.32, 95% CI 0.18 to 0.45). Mode of delivery was a significant factor in two reviews, with group-based interventions the most effective [14, 28]. A high quality review focused on women found that interventions with a group delivery component had a significant standardized mean difference of 0.36 (95% CI 0.17 to 0.54) which was 0.38 greater (*p* < 0.05), than individual or whole- of- community delivery [28]. Physical activity studies with women only did not seem to vary widely in effectiveness from those with a mixed sex sample [13]. Based on the existing evidence concerning the effectiveness of group-based interventions, these results are therefore likely to also apply to men.

There was also some evidence that intensive interventions were more effective, a medium quality review of 27 studies across all age groups [14] found that frequent facilitator contact tended to be associated with effectiveness. Similarly, a low-quality review which included six relevant interventions [27], found a pattern for successful education and support interventions when interventions initially included a more intensive phase that was either individual or group-based followed by a less intensive phase that often included individual telephone support or support groups. There was mixed evidence relating to the duration of interventions; one medium quality review concluded that interventions delivered over longer time periods were more effective [14], while another, high quality review, concluded that intervention duration did not impact on effectiveness [28]. Other factors that were associated with higher effectiveness were: the involvement of the community in the design and implementation of interventions [21]; developing community infrastructure (e.g., through sustainable partnerships) to sustain effective interventions [21]; interventions delivered through personal contact; and tailored interventions [27, 31]. There were several intervention characteristics which were not associated with the effectiveness of interventions; for example one review showed that there was no significant between-group differences for physical activity measure (objective, valid/reliable self-report, not valid/reliable self-report), delivery channel (face-to-face, telephone, mass media, print), setting (in the home, through an organisation/center, or at the broader community level), mean age of participants, or risk of bias, or the number of behavioural techniques [28]. The use of theory was inconclusive, one review found that the use of theory was associated with effectiveness [14], while another showed that there were no differences in outcomes between studies that used theory and those that did not [28].

Several reviews reported that the methodological quality of original studies was low and the risk of bias was high [14, 28] or that methodological quality was variable with some risk of bias [13]. Methodological problems that were commonly identified included the methods of recruitment, low response rate, and high rates of attrition [14, 21, 23].

#### Older adults

Notably, only one of the included reviews focused on older adults (50+) [9]. This recent, medium quality, review of community and home-based interventions, included three relevant studies, and showed that effectiveness of interventions did not differ according to level of education [9]. Thus, the review concluded that tailored print letters with feedback on current physical activity plus tailored environmental information were effective whereas similar web based interventions were not effective; educator-led chair exercises, encouragement of walking, and using a pedometer for self-monitoring were also deemed effective among older adults with low levels of education.

#### All age groups

Two reviews focused on all ages and did not provide separate outcomes according to age groups [22, 24]. One of these reviews was conducted 10 years ago, of low quality, and included 14 relevant studies [22] and the other was published in the past 5 years, of medium quality, and included 12 relevant studies [24]. Chaudhary and Kreiger [22] found that experiential activities (e.g., group exercise and interactive videos) have been successfully used as strategies to overcome barriers to health behaviour change. Further, incentives can influence uptake of physical activity and potentially have lasting impacts on physical activity attitudes and behaviour. Magnée et al. [24] found that overall, interventions had small or modest effects on physical activity and there was evidence to support community settings as the most effective intervention setting for socioeconomically disadvantaged groups. The review also indicated that intensive interventions are most likely to reduce socio economic status inequalities in physical activity.

## Discussion

In this umbrella review, we found the evidence of effectiveness of interventions varied depending on the age group examined. Among pre-school and school-aged children, there was evidence of the effectiveness of interventions; few studies focused on adolescents and interventions in this age group were generally not effective; among adults there was mixed, and thus inconclusive, evidence of effectiveness; and few studies focused on older adults and evidence was thus inconclusive. Across all age groups, the longer-term effectiveness of interventions was rarely reported and there were a range of methodological limitations, particularly in relation to recruitment and retention of participants. Where possible, we examined the characteristics of effective interventions. However, our ability to achieve this was limited because, as noted in several reviews, often interventions were insufficiently described or included multiple components. We recommend that future studies provide adequate and detailed descriptions of interventions so that the characteristics associated with effectiveness can be identified.

For preschool children, parent-focused family-based interventions in community settings were effective in improving physical activity, although these studies were of low quality and there was no indication of longer-term outcomes. Nevertheless, the following features of parent-focused interventions were associated with effectiveness in improving their preschool child’s physical activity: intensive interventions with many contacts over a longer period; group-based sessions; educational approaches; high levels of parental engagement; use of behaviour change techniques (such as goal setting); a focus on skill building not just knowledge acquisition and links to community resources to support physical activity. Although family-focused interventions were successful and we thus recommend that their long- term outcomes are assessed in future research, current studies had methodological limitations including selection bias, low recruitment rates and high attrition. Several reviews that focused adult physical activity [13, 14, 21, 23, 28] reported similar issues relation to recruitment and retention of participants. Therefore, we recommend that future studies that focus on people from socio economically disadvantaged groups consider the acceptability of interventions and the recruitment and retention of participants.

Recruiting and retaining participants from socio economically disadvantaged groups is challenging. Evidence suggests that active and targeted recruitment [32], partnering with respected community stakeholders and organisations, utilizing well-trained study staff who are ethnically, linguistically, and culturally matched to the population of interest, and use of multiple advertising channels are associated with successful recruitment of underserved population groups [33]. Strategies associated with increased retention include designing the intervention (and control condition) to be as appealing as possible with consideration of cultural tailoring and by making the intervention highly interactive; ensuring efficient tracking of participants; persistence; and demonstrating a positive, caring attitude towards participants [33]. Thus, we recommend that future studies implement these strategies to improve recruitment rates and retention in studies [34].

We found that school-based interventions were effective in improving physical activity among children from socioeconomically disadvantaged groups. School-based interventions are likely to be effective when they are embedded into school curriculum, include enhancements to physical education (e.g., requiring students to undertake physical education throughout their schooling), additional physical activity opportunities, school self-assessments of their policies, facilities and programs, and teacher, parent and student physical activity education. Given the strong evidence of the effectiveness of school-based interventions and policies, a focus on implementation and factors that influence implementation of these interventions and policies is warranted. For example, many countries have policies and regulations for the inclusion of physical education in primary and secondary schools, however, evidence suggests that compliance with this requirement is poor [35]. A study in the US showed only 46% of the districts included in the study were in compliance with physical education mandates [36]. Further, an Australian study found that 30% of government primary schools do not provide the mandated hours of planned physical activity each week [37]. The evaluation of strategies to improve compliance with physical education policies and mandates, such as public disclosure of physical education data, which has been found to increase physical education policy adherence [38], is warranted. We also found that evidence relating to the effectiveness of physical activity interventions for children and adolescents in community-based settings other than schools is scarce. Therefore, an examination of the role and effectiveness of physical activity interventions in other community-based settings, such as sporting clubs, parks and open space, and community fitness centres, is needed.

Data suggests that adolescents from socio economically disadvantaged groups experience a steeper decline in physical activity [39]. However, we found that only a small number of studies, mostly of high quality, have focused on improving physical activity among adolescents from socio economically disadvantaged groups. Of these studies, few were effective at improving physical activity. Group-based interventions were generally not effective, and thus other approaches should be examined. It has been reported that promising strategies may include involving adolescents in the development and delivery of physical activity interventions (empowerment and engagement) and involving family [20, 26]. There is however a lack of physical activity interventions targeting adolescents from socio economically disadvantaged backgrounds, therefore more research in a range of settings (e.g., school, community) is needed.

For adults, there was mixed evidence about the effectiveness of interventions and longer-term outcomes were seldom reported. However, interventions that included a group-based component were shown to be effective in improving physical activity, at least in the short term. Group education meetings, group-based physical activity sessions, or a combination, facilitated by a trained educator, health worker or practitioner were effective. The social support provided in group settings is expected to at least partly explain the effectiveness of group-based interventions, as social support is associated with physical activity among people from socioeconomically disadvantaged groups [40, 41], particularly women [40]. We recommend that future studies of adults from socio economically disadvantaged groups focus on group-based interventions and examine the long-term outcomes of such interventions.

For adults, interventions that focused on physical activity only, rather than multiple behaviours, were likely to be more effective. A similar finding was reported in a previous umbrella review [42], which focused on the effectiveness of single and multiple behaviour change interventions in improving physical activity and dietary behaviours. There are several potential explanations for this finding. Interventions that target multiple behaviours may be less successful than single behaviour interventions due to the nature of the intervention itself. A systematic review of obese adults found that single behaviour change interventions and multiple behaviour change interventions differed in the numbers and type of behaviour change strategies that are used [43]. Further, individuals may find it difficult to change several behaviours at once. According to the ego depletion model, self-regulation draws on limited resources [44] which may be best applied to one behaviour change target at a time. Finally, interventions that target multiple behaviours are likely to be less intensively focused on physical activity and, as previously discussed, more intensive interventions tend to be more effective in improving engagement in physical activity. Conclusions about the effectiveness of single compared to multiple behaviour change interventions must be interpreted with caution, however, because the included reviews compared the results of single behaviour change interventions with the results of multiple behaviour change interventions, and did not specifically examine studies that included comparisons of single versus multiple behaviour change interventions within the same study [42]. We recommend that future studies examine the differences on behavioural outcomes between interventions that target single behaviours (i.e. physical activity only) and multiple behaviours (e.g., diet and physical activity) in a single study to provide more robust guidance on effectiveness in socioeconomically disadvantaged groups.

There have been few studies that examine the effectiveness of interventions among older adults from socio economically disadvantaged groups. We identified one review, which included three studies that examined the effectiveness of interventions according to level of education. Based on the limited available evidence, tailored printed informational-material, group exercise programs, which incorporated self-monitoring through devices such as pedometers show promise in improving engagement in physical activity among this group [9]. Given that few studies have been conducted with older adults, we recommend that more research be conducted on the effectiveness of interventions for improving physical activity among older adults for socio economically disadvantaged population groups. Such interventions should include promising strategies such as tailored information and self-monitoring.

Across all age groups, we found that interventions that were more intensive (e.g., more contacts), were more successful at improving engagement in physical activity than less intensive interventions. However, because few studies examined long-term outcomes, there is uncertainty about whether the changes in physical activity from intensive interventions are sustained over time. Interestingly, there was mixed evidence relating to whether or not the duration of the interventions was associated with effectiveness. People from socioeconomically disadvantaged groups face multiple barriers to engagement in physical activity, therefore intensive interventions may be necessary to successfully change behaviour [45]. However, because intensive interventions are more expensive and demanding [46], their sustainability is questionable. Alternatively, low-intensity interventions may be less efficacious, but have the potential to be delivered widely across the community or across many groups [47]. This is particularly pertinent, given that even small increases in physical activity could benefit population health, with one study suggesting the largest gains come from inactive individuals becoming moderately active [48]. We thus recommend that future studies consider the sustainability of intensive interventions and also consider the broader population health impacts of interventions when assessing their effectiveness.

Seven of the 17 reviews (41%) focused on obesity prevention (but included physical activity outcomes) [15, 17, 20, 24, 25, 29, 30] . We also excluded an additional seven reviews, at the full text review stage, that were focused on obesity prevention but did not report physical activity outcomes [49–55]. The focus of these reviews perhaps reflects the attention of public health and medical researchers on obesity prevention and treatment, rather than lack of physical activity [56]. This is despite evidence that low physical activity participation is comparable to obesity as a risk factor for poor health outcomes [41]. It has also been argued that a focus on obesity prevention and treatment, rather than the numerous other benefits of physical activity, could discourage many people from engaging in physical activity [57]. As engagement in physical activity was either not the main outcome examined in several reviews or was one of several outcomes that were examined, it was difficult to identify the components of effective interventions specifically for physical activity. We recommend that future studies consider the broad range of benefits of physical activity, as this may have implications for the design and nature of interventions. We also recommend that future systematic reviews focus on participation in physical activity, to provide more detail about the effectiveness of interventions for improving physical activity as well as the characteristics of effective interventions.

The study designs of primary studies that were included in the reviews were often difficult to determine, due to the use of vague descriptions such as ‘any intervention’. Nevertheless, the reviews included in our umbrella review comprised a range of study designs, with one [13] being limited to RCTs only. In the context of physical activity interventions, the inclusion of a broader range of study designs, such as natural experiments, is appropriate and provides a more comprehensive assessment of existing evidence than limiting reviews to RCTs only [58]. RCTs are considered to be the ‘gold standard’ for demonstrating a causal link between an intervention and an effect [59], however, acceptance of RCTs as the gold standard source of evidence may limit the knowledge base needed to make sound decisions about public health priorities and policies. It has been argued that, for evaluating large-scale interventions, studies with quasi-experimental designs (such as natural experiments) are often the only feasible option and may provide valid evidence of impact and provide evidence that is more ecologically valid [58]. Thus, the inclusion of a broad range of study designs when examining physical activity interventions is warranted, particularly when examining large-scale physical activity interventions, which are usually multi-component and their pathways to impact are complex and subject to effect modification and where a RCT design may be neither feasible nor ethical [60, 61].

### Strengths and limitations

Limitations intrinsic to umbrella reviews also apply to our review. These include potential overlap in studies included in evidence syntheses [62]. We found that several studies were included in more than one review and adopted a position similar to Green et al. [63], concluding that the precise degree of overlap of studies between reviews was difficult to ascertain and was not central to the objectives of this review. It should also be noted that most of the reviews included in our umbrella review were of low quality; out of 17 included reviews, less than one third were rated as high quality. The main methodological problems noted for the reviews were: (1) failure to conduct a test of publication bias (or at least note that assessment was not possible due to less than 10 included studies), (2) failure to include grey literature as a source of information and (3) failure to include a list of excluded papers. Fourteen reviews scored a zero for all 3 above criteria and no review scored a point for including a list (or reference to) excluded papers. However, whether these items reflect methodological quality or merely reporting quality is debateable and some items are difficult to interpret and apply consistently [64]. Furthermore, criterion rated as ‘not applicable’ are scored the same as ‘No - not assessed/reported’ thereby biasing non-meta-analyses to lower overall scores.

Strengths of our umbrella review include a comprehensive analysis of reviews of a range of physical activity interventions across all age groups. Along with an examination of the effectiveness of interventions and identification of characteristics associated with the effectiveness of interventions (albeit with limitations already discussed) we have outlined recommendations for future research across each age group to provide directions for future research.

## Conclusion

People from socio economically disadvantaged groups are far less likely to achieve recommended levels of physical activity and are more likely to experience poor health outcomes, than those from less disadvantaged groups [3, 16]. It is thus important that efforts focus on improving physical activity in this group. In this umbrella review, we found that interventions were successful at improving physical activity among children, there was mixed evidence of effectiveness for adults, and few studies have focused on adolescents or older adults. The characteristics of effective interventions are not clear, which limits the ability to make recommendations about ‘what works’ for this population group. Physical activity interventions that are designed with a broad range of benefits in mind, not just obesity prevention, should be trialled. Such interventions may lead to innovative and integrated approaches to improve physical activity that appeal to people from socio economically disadvantaged population groups. Finally, we recommend that future studies include additional indicators besides effectiveness to determine the public health impact of interventions, using frameworks such as RE-AIM, which incorporates Reach, Effectiveness, Adoption, Implementation and Maintenance [47].

## Additional file

Additional file 1: Data extraction table. Study characteristics of selected reviews. (DOCX 59 kb)

## Abbreviations

BMI: Body mass index
RCT: Randomized controlled trial
SMD: Standardized mean difference
