# Supplementary material for: Interventions to improve physical activity among socioeconomically disadvantaged groups: an umbrella review
Source: Int J Behav Nutr Phys Act. 2018 May 15;15:43. doi: 10.1186/s12966-018-0676-2 (PMC5952843; doi:10.1186/s12966-018-0676-2)
Supplement: Supplementary file 1 — Data extraction table. Study characteristics of selected reviews. (DOCX 59 kb) [file 12966_2018_676_MOESM1_ESM.docx]

# Additional File 1: Data Extraction Table

| **Author** | **Review Aim** | **Sample / Target Group** | **Disadvantaged definition** | **Included studies n(N)** | **Study designs** | **Settings included (n)** | **Intervention components** | **Main results** | **Evidence and Conclusion** |
| --- | --- | --- | --- | --- | --- | --- | --- | --- | --- |
| Bock et al. 2014 [31] | To investigate the effectiveness of community-based physical activity interventions by mode of delivery, study quality and to analyse intervention effectiveness in different subgroups in the population | Adults (18+ years) from "developed countries"; subgroup defined as "persons with low SES". | No definition provided for low SES. | 8 (55) | RCT or quasi-experimental study with comparison group. | Community (8) | Not reported for low SES | 4/8 studies on persons with low SES reported positive PA changes although the mean % change was low and insignificant (7.7% [−6.7%; 22.0%]; p = 0.248) | Information on those with low SES was generally too limited to reach a clear conclusion. |
| Bull et al. 2014 [13] | To conduct a systematic review and meta-analysis examining the effectiveness of behavioural interventions targeting diet, physical activity or smoking in low-income adults. | Low income adults (18+ years). | Studies had to explicitly refer to participants as "low-income". | 12 [7 PA only; 5 PA + diet] (35) | RCT and cluster-RCT | No data specific to PA studies | Tailored self-help materials (1); education/tailored feedback (4); individual counselling (4); group programmes (1), structured exercise (4) | Post-intervention effects were positive but small for PA (SMD 0.21, 95% CI 0.06 to 0.36).   Follow-up results suggested that effects were not maintained for PA (SMD 0.17, 95% CI −0.02 to 0.37).  Subgroup analyses for heterogeneity suggested SMDs were not different (p=0.48) in four interventions targeting women only (SMD 0.14, 95% CI 0.00 to 0.27) compared with eight with a mixed sex sample (SMD 0.24, 95% CI −0.02 to 0.49). Effects were larger (p<0.001) in the 7 interventions targeting PA only (SMD 0.32, 95% CI 0.18 to 0.45) than ﬁve interventions targeting multiple behaviours including physical activity (SMD 0.00, 95% CI −0.07 to 0.08) | PA behaviour change interventions for low income groups had small positive effects.   Overall, studies did not describe intervention contents comprehensively, making 'what works' difficult to ascertain.  The quality of studies was variable with some risk of bias identiﬁed however no association between study quality and outcomes explored. |
| Chaudhary & Kreiger 2007 [22] | To develop an inventory of community-based nutrition and physical activity intervention strategies for chronic disease prevention in low-income populations. | Healthy, free-living participants of any age who were defined as low-income. | The low-income parameter was defined "either explicitly by the authors or implicitly by subject enrolment in programs for low income clients". | 14 (41) | Any intervention | Community (14)  (NB. broad definition used - i.e. not institutional or controlled environments) | Interventions ranged from traditional group sessions taught by allied health professionals, peer-led workshops, one-on-one counselling (n=5), print material, and home-based telephone and mail interventions to multimedia strategies and experiential activities such as group exercise. | Results suggested that “PA interventions aimed at low-income audiences tend to be delivered in an interactive visual format, to be culturally appropriate, to be administered in accessible primary care settings, and to give incentives to act on the information provided." "The majority of interventions incorporated conceptual frameworks or were guided by theories. Where applied, these theories were tailored to the participants' conceptual development." | Experiential activities such as group exercise and interactive videos have been used as strategies to overcome barriers to health behaviour change.  One-on-one behavioural counselling by PHC professional suggested as "ideal" with the advent of interactive multimedia and home internet as cost effective and similarly impactful.  Incentives can act as "short-term luring mechanisms" but may also effect "lasting changes in PA attitudes and behavior". |
| Cleland et al. 2012 [14] | To examine the effectiveness of interventions to promote physical activity in socio-economically disadvantaged communities; to identify the theoretical frameworks and components of effective interventions. | All ages; studies targeting socio-economically disadvantaged communities | Defined as an "area, neighbourhood or community with residents clearly defined as disadvantaged, relative to the wider national population". Definitions could relate to income, educational level, ethnic diversity or public housing. | 27 (27) | Any intervention | No information provided | Studies targeted communities (5), individuals (4) or groups (18).  Interventions were mostly multi-component including: counselling, problem solving, behaviour change strategies, education, physical activity and social support, combined with professional support. | Overall, 14/27 interventions were found to be effective. Individuals: 3/4 studies showed negligible or small effects on PA (SMD 0.43, 95% CI -0.37 to 1.23]). Groups - Adults: 6/12 studies showed a moderate (2) or small (4) effect of PA. The remainder showed no significant effect or were insufficiently detailed to determine their effect (SMD 0.36, 95% CI 0.06, 0.65). Groups - Children: 2/6 group interventions targeting children and adolescents showed significant effects (SMD 0.59, 95% CI -0.77, 1.95). Community: 3/5 interventions had a small effect and 1 had a moderate effect on PA. | Group-based interventions were considered effective for adults but not for children; evidence for the effectiveness of interventions targeting individuals was deemed insufficient; limited evidence suggested that community-wide interventions produced small changes in PA. Interventions underpinned by any theoretical framework, compared to none, were more likely to be effective. Furthermore, frequent facilitator contact, over longer periods, tended to be associated with effectiveness.  Methodological problems of studies with low SES populations included sample identification, inclusion criteria and methods of recruitment. High rates of attrition noted. |
| Cleland et al. 2013 [28] | This study aimed to determine the effectiveness of interventions to increase physical activity among women experiencing disadvantage, and the intervention factors associated with effectiveness. | Women aged 18-64 years; socioeconomically disadvantaged | Socioeconomic disadvantage defined as those with low education, low income, unemployed, low status occupations or living in an area of low socioeconomic status. | 19 (19) | Controlled interventions | Community (n=12); Organisation/Centre (n=6); Home (n= 1) | Delivery via community (3), individuals (5) and groups (11).  Most commonly reported behaviour change techniques: providing information about behaviour–health links (n = 12), prompting of barrier identification (n = 11), and planning for social support or social change (n = 10). | Studies with a group delivery component had a significant SMD of 0.36 (0.17, 0.54) which was 0.38 greater (p<0.05) than both individual or community-based delivery.  No significant between-group differences was observed for any other variable including: PA measure, delivery channel, setting, intervention duration, age group use of theory or type of theory used. | Programs with a group delivery mode significantly increase physical activity among women experiencing disadvantage. Group delivery should be considered an essential element of PA promotion programs targeting this population group.  Most studies used self-reported measures of PA, and the risk of bias was high – only 5 of the 19 studies were deemed to have medium or low risk of bias |
| Everson-Hock et al. 2013 [23] | To assess the effectiveness and acceptability of community-based dietaryand physical activity interventions among low-SES groups in the UK. | Adults (aged 18–74) from a low-SES group within the UK. | No definition. | 4 [2 PA and 2 PA+other] (35; 12 quantitative and 23 qualitative) | Any intervention (also included qualitative evaluations of interventions assessing PA beliefs among low SES and health professionals working with low-SES) | Community (4) | Community awareness campaign (1), tailored exercise plan plus counselling and vouchers (1), behaviour change plus education and empowerment (1), internet-based education and peer-support (1) | Quantitative: Overall, PA-focused interventions showed mixed effectiveness with one study finding a positive effect and another finding a mixed effect. For multicomponent interventions, no significant impact was seen for PA.  Qualitative: sufficient available resources deemed important for implementation; fear of crime, intimidation and attack, dark evenings and poor weather were barriers to outdoor physical activity. | The effectiveness of community-based physical activity interventions is inconclusive. A range of barriers and facilitators exist, some of which were addressed by interventions but some of which require consideration in future research. |
| Kader et al. 2015 [30] | To review: 1) effectiveness of universal parental support interventions to promote dietary habits, physical activity (PA) or prevent overweight and obesity among children 2–18 years and 2) effectiveness in relation to family socio-economic position | The study included at least one parent or caregiver of a child 2–18 years, either with or without their child | Definition of low SEP or belonging to a minority group was based on the original authors' definition. | 3 (35) | Any controlled intervention where the main component was parental involvement. | Community (2), Pre-school (1) | Group education/training (3). | 2/3 studies with parents of pre-school children from low SEP groups were effective in improving PA, with the remaining study showing no effect and a high drop- out rate. | In groups with low socio-economic position, intensive, group-based educational approaches appeared promising but low participation rates and high attrition was a problem to consider.  Most studies were deemed moderate to low quality. |
| Kornet-van der Aa et al. 2017 [20] | To summarize the evidence on the effectiveness of obesity prevention and treatment programmes for adolescents from socioeconomically disadvantaged backgrounds. A secondary aim to identify potential successful intervention strategies for this target group. | Adolescents (12-18 years) from disadvantaged backgrounds | Living in low-income communities or attending schools situated in low-income areas, or when a stratified analysis for SES was performed. | 5 (9) | Any obesity intervention (controlled trials and pre-post studies without control) | School (4), healthcare (1) | All interventions were multi-component, combining behavioural, educational and/or environmental components, except for one media-based educational intervention | None of the 5 PA studies (all moderate-high quality) reported significant effects.  Authors found no conclusive evidence for which specific intervention strategies were particularly successful in preventing obesity among disadvantaged adolescents. | Community-based interventions were not successful in improving adolescent physical activity from disadvantaged backgrounds. |
| Laws et al. 2014 [15] | To examine the effectiveness of interventions to prevent obesity or improve obesity related behaviours in children (0-5 years) from socioeconomically disadvantaged or Indigenous families. | Children (0-5 years) from socioeconomically disadvantaged backgrounds or indigenous families. | Socioeconomically disadvantaged families and their children defined as those described as low socioeconomic status, low income, low education (high school or below), or from low income areas. | 11 (32) | Any | Community (n = 2), home based (n=2), health care setting (n = 2), preschool (n = 5), | Delivery agent: paraprofessionals (2), nurses (1), dietician (1), PHC staff (1), Child educators/teachers (5), Researchers (1). | Of the 11 studies listed with a PA outcome, 6 were classified as showing significant effect and 5 with no effect. All studies targeted multiple health behaviours. For the 3 studies where improving child PA behavior was primary aim, all reported significantly positive PA effect. Both community setting based interventions were graded as positive. | Most studies were low or moderate quality and did not use validated measures, and/or had high probability of selection bias. |
| Lehne and Bolte 2017 [9] | To analyze whether and how studies of interventions consider effects on social inequalities in PA among older adults | General population of older adults (50+ years) | Data on social factors were considered via the PROGRESS-Plus framework (including SES, income and education) for measuring intervention. | 3 (11) | Any intervention | Community (1), Home (2) | Educator-led exercise classes with self-monitoring (1), tailored letters with information feedback (2), web-based tailored information and feedback (1) | 3 studies in older adults examined differential effects based on education status. All 3 interventions were positive for PA change (one was mixed depending on intervention arm) and no significant interaction effects were found for education in any study. | Interventions were similarly effective (or ineffective) regardless of education in older adults. Tailored print letters with feedback on current PA plus targeting of environmental determinants were effective; web-based interventions not effective; educator-led chair exercises, encouragement of walking, and using a pedometer appear to be effective. |
| Magnee et al. 2013 [24] | To investigate differential effectiveness of interventions aimed at obesity prevention, the promotion of PA or a healthy diet by SES. Re-analysis of Dutch obesity-related lifestyle interventions. | Dutch population, any age, with a measured indicator of SES. | Any indicator of SES [Educational level was used as indicator of SES in 25 studies]. | 12 (26) | Any | Community (4), workplace (3), school (1), healthcare (1), Individual (3) | Could not be extracted | For PA-focused interventions, 1 study decreased inequality, 4 were neutral and 2 increased inequalities. The same pattern was seen for combined PA plus diet interventions. | Two/four community studies provided evidence for better effectiveness in lower-SES groups; 2 others were neutral. One high-intensity community-based study provided best evidence for higher effectiveness in low-SES groups. Workplace and individual focused studies were either neutral or more effective in high SES groups. |
| Olstad et al. 2016 [25] | To examine the impact of universal policies on socioeconomic inequities in obesity-related behaviours (including PA) among adults and children. | All ages [children (0-17) and adults (18+) considered separately]; analysis according to socioeconomic position | A subgroup of participants had to be disadvantaged, defined by one or more eligible measures. Eligible measures included income, education, occupation, participation in low-income support programmes, area or setting-level disadvantage, other indicators of material resources such as car ownership or housing tenure, and aggregate indices." | 7 [4 with children; 3 with adults] (36) | Any quantitative design. | Community (2), School (3), Organisation (1), Other (1). | Policy: structural (2), agento-structural (4), mixed (1); microenvironmental (4), macroenvironmental (3) | 2/7 policy studies rated as positive for reducing PA inequity, 1 rated as mixed impact and the remainder as neutral. For the two positive studies, one was categorised as Structural/Microenvironmental (a provincial school PE policy) and the other as Agento-Structural/Macroenvironmental (children's fitness tax credit) policy. | Overall, for all obesity-related behaviours, no clear patterns of policies that positively or negatively impacted inequities could be discerned with most universal polices having a neutral impact. Notably, no policies negatively impacted inequities in PA behaviour. |
| Olstad et al. 2017 [17] | To examine the impact of targeted policies on obesity-related behaviours amongst socioeconomically disadvantaged children and adults. | Adults (≥18 years) or children (≥ 2 years); socioeconomically disadvantaged populations | Socioeconomically disadvantaged individuals in a developed nation, or all individuals within a disadvantaged setting. | 6 (18)  Children/adolescents only (4), adults only (1), parents and children (1) | RCTs, quasi-experimental controlled pre–post studies and regression discontinuity designs. | Community (1), School (4), Healthcare clinic (1) | Organisational policy (3), Government policy (3)  All organisational policies were part of multi-component school-based interventions. All government policies were single policy interventions. | Overall, 2/6 policy containing interventions were effective for PA change in children (one organisational and one governmental) with one being effective for children but not parents. One organisational policy intervention aimed at schoolchildren had mixed results being null for accelerometry outcome but positive for sport participation. The remainder had no PA impact. | Overall, effectiveness was suggested for comprehensive interventions that included school policies, and for government policies targeting children in school settings. "Interventions during childhood may ameliorate negative obesity-related manifestations of socioeconomic disadvantage. Gaps in knowledge remain surrounding effective policies in adults, adolescents and very young children."  The majority of studies received strong or moderate quality ratings and were conducted over the longer term. |
| Taylor et al. 1998 [21] | To summarize interventions that have targeted populations at risk for inactivity. | Adults (21+ years); Low-income, racial and ethnic minority, and populations with disabilities | People with low incomes, members of some ethnic minority groups, and those with disabilities. | 10 (14) *NB only 8 studies with actual PA change outcomes* | Any PA intervention | Community (4), community + home (1), church (1), school (1), home (1), | Interventions included: self-change strategies (1), exercise classes (6), walking clubs (1), education (3), counselling (2), groupeducation/support (4). Most were multicomponent. | Out of 8 studies with PA data, only 2 interventions reported consistent and positive PA changes); 2 showed mixed results and 2 were positive for specific sub-groups only (e.g. community coalitions or organised communities).  Common intervention features for the 10 studies that included low-income/ethnic minority groups were community advisory panels, community needs assessments, and community members delivering the intervention. Eight studies reported a theoretical framework that guided the intervention. | Involving the community at all steps in the design and implementation of the intervention shows greatest promise for promoting behavior change.  Authors noted various challenges for successful interventions including: use of a theoretical framework, valid and reliable measures, a  strong experimental design, an effective intervention, and minimal attrition. |
| van Sluijs et al. 2007 [26] | To evaluate the effectiveness of interventions to promote physical activity in children and adolescents. | Children (<12 years) and adolescents (≥12-18 years). | No definition provided | 5 [Children=3, Adolescents=2] (57) | Any intervention | School (2), School + Other (3) | Multi-component = 2, Educational = 3 | Children PA interventions (n=3): All 3 controlled trials targeting low SES children reported significant positive effects, resulting in a classification of limited evidence of an effect. Adolescent PA interventions (n=2): Only one of two intervention studies aimed at low socioeconomic adolescents reported a significant intervention effect. Consequently, evidence of an effect was deemed inconclusive. | For children with low SES backgrounds, some evidence of effect was observed based on mostly high-quality studies  For adolescents the evidence was deemed inconclusive based on small number of otherwise high-quality studies.  Limitations across  the studies included short duration of follow-up, inadequate adjustment for potential confounders, and a lack of adjustment for clustering when randomisation was carried out at group level. Another limitation was the lack of precision of the PA outcome measures. |
| Walton-Moss et al. 2014 [27] | To critically review community-based cardiovascular disease (CVD) interventions to improve cardiovascular health behaviors and factors among vulnerable populations. | All ages; vulnerable populations. The included low SES studies all in adults | Vulnerable populations included racial and ethnic minorities, those of low SES or low literacy, and individuals who reside in geographic isolation or poverty. | 6 (32) | Any community-focused intervention | Community (6) | Data could not be extracted | In low income/low SES populations (n=6 studies): 2 studies improved MPA and/or VPA (with one being significant for self-report measures only); 1 study increased walking volume. One study showed an improvement at 6 weeks (but a significant reduction at 12 weeks post-intervention) | PA interventions "appeared to be more efficacious for low-income or individuals living in socially disadvantaged communities again, regardless of race or ethnicity."  There was a "pattern for successful education and support interventions with often initially a more intensive phase that was either individual or group based followed by a less intensive phase that often included individual telephone support or support groups." |
| Wijtzes et al. 2017 [29] | To evaluate the effectiveness of interventions aimed to improve lifestyle behaviours and/or prevent overweight among socially disadvantaged children in Europe | European children ≤12 years; socially disadvantaged | Socially disadvantaged children were defined as children with a non-native ethnic background/immigrant status or children from families with a low socio-economic status (i.e. low parental educational level, low household income, low parental occupational class, or living in low income/deprived areas)" | 5 [4 interventions] (11 studies evaluating 8 interventions) | Controlled trials (concurrent control group) | School/preschool (5) | Cultural tailoring in 2/4 interventions with another including language specific parent information.Interventions included: child education (2), structured exercise (4), parental group education (2), environmental modification (1), behaviour change strategies (1) | All 3 PA-focused (i.e. primary outcome) interventions in primary school aged children were deemed effective. By contrast, neither of the two weight prevention-focused interventions were effective for PA change (secondary outcome). Furthermore, only 1/4 studies with objectively measured PA showed a significant, positive effect (increased daily steps). | Interventions targeting one specific behaviour (e.g. PA) were moderately effective in changing that behavior. |

# Abbreviations:

# PA = physical activity; SMD = standardized mean difference, RCT = randomized controlled trial; SES = socio-economic status; CVD = cardiovascular disease; MPA moderate-intensity physical activity; VPA vigorous intensity physical activity
